# Transient loss of consciousness caused by cryptococcal meningitis in an immunocompetent patient: a case report

**DOI:** 10.1186/1757-1626-2-60

**Published:** 2009-01-15

**Authors:** Lee Wilbur, Ryan Heyborne

**Affiliations:** 1 Indiana University; Department of Emergency Medicine, 1050 Wishard Blvd; Indianapolis, IN 46202, USA; 2 Emergency Medicine and Trauma Center/Methodist Hospital, Department of Emergency Medicine; Indianapolis, IN 46202, USA

## Abstract

An immunocompetent 51 year-old female presented to the Emergency Department (ED) with a chief complaint of transient loss of consciousness and was found to have cryptococcal meningitis. She complained of general fatigue and a 'cramping' sensation in her right arm for one week. The physical exam was significant for the presence of a right homonymous hemianopsia and the absence of fever or signs of meningismus. A computed tomography (CT) of the brain was interpreted as showing an age-indeterminate infarct in the left parietal region. However, a magnetic resonance imaging (MRI) of the brain showed multiple areas of meningeal enhancement. Cerebrospinal fluid analysis revealed a positive cryptococcal antigen and 105 white blood cells. The patient was successfully treated with Amphotericin B and fluconazole. While cryptococcal meningitis is typically a disease of the immunocompromised, it can, as in this case, present in an immuncompetent host.

## Introduction

Transient loss of consciousness is a common complaint representing 3–5% of ED visits annually in the United States [1]. The differential diagnosis for transient loss of consciousness is broad and includes meningitis. In this case the likelihood that this patient suffered from meningitis was low given the fact that she was afebrile, immunocompetent, and without meningismus. Cryptococcal meningitis, caused by the encapsulated yeast Cryptococcus neoformans, is known most notably as an opportunistic infection in patients with human immunodeficiency virus (HIV) and other conditions associated with an immunocompromised state (alcoholism, diabetes, malignancy, etc.). Recently, reports of cryptococcal infections in immunocompetent hosts have been described in the literature [2-6]. The initial clinical presentation of these patients have included pulmonary infections, sepsis syndromes, and subacute meningoencephalitis. To our knowledge, this is the first reported case of cryptococcal meningitis in an immunocompetent patient presenting to the ED with transient loss of consciousness.

## Case presentation

A 51-year-old woman presented to the ED with the chief complaint of transient loss of consciousness and right arm pain while washing dishes. She was unable to recall the amount of time she lost consciousness but stated that she was at her baseline mental status when her daughter returned home from work that evening. In the ED, the patient denied chest pain, palpitations, abdominal pain, or vertigo prior to her inciting event. She stated that she had noticed an increase in general fatigue and headaches for two weeks, but denied fever, myalgias, or any sick contacts. She acknowledged that her headaches felt like her migraines except that they had been constant for one week. The patient described her arm pain as an intermittent cramping sensation that had been present for less than twenty-four hours. Her past medical history was significant for migraine headaches, chronic hepatitis C, congestive heart failure, chronic obstructive pulmonary disease, and a recent diagnosis of a urinary tract infection. Medications included ciprofloxacin, propranolol, furosemide, spironolactone, albuterol, trazodone, and esomeprazole. Her social history was significant for a remote history of intravenous drug use and daily alcohol use until 7 months prior to presentation. Family history was significant for coronary artery disease and diabetes. The patient denied any medication allergies. Presenting ED vital signs were a temperature of 98.0 degrees, blood pressure 117/81 mm Hg, pulse 76 beats/min, respirations 18 breaths/min, and oxygen saturation of 98% on room air.

The physical exam revealed a patient in no apparent distress that was alert and oriented to name, place and time. She had equal and reactive pupils, a normal oropharyngeal exam, supple neck without pain on range of motion, clear lung fields, a normal cardiac and abdominal exam, and normal extremities. Her skin did not show any signs of a rash or stigmata of chronic liver disease. Neurologic testing revealed a right homonymous hemianopsia, normal strength, deep tendon reflexes, and sensation bilaterally. Her gait was steady and she had a normal cerebellar exam.

A CT of the brain demonstrated an age-indeterminate infarct in the left parietal region and multiple areas of new small vessel ischemic disease. Laboratory evaluation revealed a serum white blood cell count (WBC) of 8.9 × 10^9 ^with 78% neutrophils, 16% lymphocytes, 4% monocytes and 1% eosinophils, a hemoglobin of 13.8 gm/dl, and platelets 88 k/cumm. While her basic electrolytes were unremarkable, the liver function panel revealed a total bilirubin of 3.5 mg/dL, alkaline phosphatase 100 units/L, SGOT (AST) 104 units/L, SGPT (ALT) 74 units/L, and ammonia 15 mcmol/L.

Based on these data, Neurology consultation was obtained. They admitted the patient with a presumptive diagnosis of acute cerebrovascular accident (CVA) involving the occipital region. An MRI of the brain the next morning revealed multiple areas of meningeal enhancement without evidence of CVA. A lumbar puncture (LP) was performed and the cerebrospinal fluid (CSF) revealed 105 white blood cells with 29% monocytes, 0 polymorphonuclear cells, and 71% lymphocytes, 0 red blood cells, elevated protein at 176 mg/dL, low glucose at 21 mg/dL, and the cryptococcal antigen returned positive with a titer of 1:32. Human immunodeficiency virus tests were all negative. The patient was subsequently started on Amphotericin B.

The patient's hospital course was complicated by transient acute renal failure due to Amphotericin B requiring a medication change to liposomal Amphotericin and fluconazole. The CSF culture remained negative for any growth and the cryptococcal antigen titer decreased to 1:8 and 1:2 on two subsequent LP's. The patient was discharged on hospital day 21 without neurologic sequalae and was instructed to take fluconazole 400 milligrams once daily for 24 weeks duration then 200 mg daily for life. Her cyptococcal antigen became undetectable 4 weeks following her hospital discharge.

## Discussion

Cryptococcal meningitis is a common opportunistic infection in acquired immunodeficiency syndrome (AIDS); however, its prevalence among immunocompetent hosts is not well established but may be increasing [2-6]. Potential explanations for this increase include the success of anti-retroviral therapy and a difference in virulence among varieties of Cryptococcus neoformans serotypes, namely var. neoformans, var. grubii, and var. gattii [6]. Cryptococcus neoformans var. gattii is believed to be the most virulent [7].

The clinical course of cryptococcal meningitis is indolent with a median time to diagnosis from symptom onset of 44 days with a range of 7 days to 1 year.[3] Transmission is inhalational in the majority of cases [7]. Common presenting symptoms include headache, fever, and malaise. Classic meningitic findings, such as nuchal rigidity, are absent in 75% of cases.[5] Visual changes have been reported, such as the field defects seen in our patient, as well as new onset seizure activity [8]. Cryptococcal meningitis has also been shown to cause ischemic brain injury [9].

Cryptococcal antigen detection in the CSF is 99% sensitive when titers are 1:2048 and remains the most sensitive confirmation test for establishing the diagnosis [7,10]. Computed tomography of the brain is normal in 50% of proven cases; however, a focal neurological exam and abnormalities on CT or MRI of the brain have both been linked to a poorer prognosis [7]. Mortality rates for cryptococcal meningitis in AIDS patients and immunocompetent patients are estimated to be between 30% to 50% and 25 to 44%, respectively [2,5]. Recommended treatment includes a 3 step treatment approach: induction (2 weeks of amphotericin B 0.7–1 mg/kg/day plus flucytosine 100 mg/kg/day) followed by consolidation (8 weeks of fluconazole 400 mg/day) and then maintenance (fluconazole 200 mg/day for life) [7].

The patient described in this case had a rare etiology of a common ED complaint. Clues to the diagnosis in this case included an atypical headache and fatigue for 2 weeks (subacute), ischemic changes on head CT, a homonymous hemianopsia, and meningeal enhancement on the initial MRI. The physicians' focus on a CVA as the etiology in this particular patient could have resulted in a significant delay in therapy and a poor outcome. Physicians should be made aware that cryptococcal meningitis is increasing among the immunocompetent population, and it may present as subacute meningoencephalitis without classic signs of meningismus. Precise prevalence figures are not known which may be due to under-reporting secondary to the belief that cryptococcal meningitis is typically seen in the immunocompromised population. Detection of cryptococcal meningitis in the immunocompetent host may improve if CSF cryptococcal antigen tests were sent on cases of patients suspected of having meningitis in the ED. This novel case is the first reported immunocompetent patient that was found to have cryptococcal meningitis after presenting to the ED with transient loss of consciousness.

## Conclusion

Clinicians should be informed that cryptococcal meningitis can present in immunocompetent hosts and may appear clinically dissimilar to traditional meningitis.

## Abbreviations

mmHg: millimeters of mercury; gm/dl: grams per decilitre; k/cumm: thousand per liter; AST: aspartate aminotransferase; ALT: alanine aminotransferase; units/L: units per liter; mcmol/L: micromoles per liter; mg/dl: milligrams per decilitre; mg: milligrams.

## Consent

Written informed consent was obtained from the patient for publication of this case report. A copy of the written consent is available for review by the Editor-in-Chief of this journal.

## Competing interests

The authors declare that they have no competing interests.

## Authors' contributions

LW serves as an attending physician in the hospital in which this patient was treated. He is primarily responsible for the contents of this manuscript. He provided final editing and submitted the manuscript. RH is a resident physician at Indiana University and clinically treated the patient included described in this manuscript. He contributed significantly to the content of the manuscript and has read and approved the final version.

## References

[B1] Marnolis A Linzer M Syncope: Current diagnostic evaluation and management Ann Int Med 1990 112 850 863 2188544 10.7326/0003-4819-112-11-850

[B2] Bicanic T Harrison TS Cryptococcal meningitis Br Med Bull 2005 72 99 118 10.1093/bmb/ldh043 15838017

[B3] Ecevit IZ Clancy CJ Schmalfuss IM Nguyen MH The poor prognosis of central nervous system cryptococcosis among nonimmunosuppressed patients: a call for better disease recognition and evaluation of adjuncts to antifungal therapy Clin Infect Dis 2006 42 1443 7 10.1086/503570 16619158

[B4] Imwidthaya P Poungvarin N Cryptococcosis in AIDS Postgrad Med J 2000 76 892 85 8 10644384 10.1136/pmj.76.892.85 PMC1741486

[B5] Lui G Lee N Ip M Choi KW Tso YK Lam E Chau S Lai R Cockram CS Cryptococcosis in apparently immunocompetent patients QJM 2006 99 3 143 10.1093/qjmed/hcl014 16504989

[B6] Dromer F Mathoulin-Pélissier S Launay O Lortholary O The French Cryptococcosis Study Group Determinants of disease presentation and outcome during cryptococcosis: the cryptoA/D study PLoS Med 2007 4 2 e21 17284154 10.1371/journal.pmed.0040021 PMC1808080

[B7] Sloan D Dlamini S Paul N Dedicoat M Treatment of acute cryptococcal meningitis in HIV-infected adults in resource-limited settings Cochrane Database System Rev 2006 8 4 CD005647 10.1002/14651858.CD005647.pub2 18843697

[B8] Mwanza JC Nyamabo LK Tylleskar T Plant GT Neuro-ophthalmological disorders in HIV infected subjects with neurological manifestations Br J Ophthalmol 2004 88 11 1455 9 15489493 10.1136/bjo.2004.044289 PMC1772396

[B9] Lan SH Chang WN Lu CH Lui CC Chang HW Cerebral infarction in chronic meningitis: a comparison of tuberculous meningitis and cryptococcal meningitis QJM 2001 94 5 247 253 10.1093/qjmed/94.5.247 11353098

[B10] Antinori S Radice A Galimberti L Magni C Fasan M Parravicini C The role of cryptococcal antigen assay in diagnosis and monitoring of cryptococcal meningitis J Clin Microbiol 2005 43 11 5828 5829 16272534 10.1128/JCM.43.11.5828-5829.2005 PMC1287839

